# Rice dwarf virus infection alters green rice leafhopper host preference and feeding behavior

**DOI:** 10.1371/journal.pone.0203364

**Published:** 2018-09-07

**Authors:** Qianjin Wang, Jingjing Li, Cong Dang, Xuefei Chang, Qi Fang, David Stanley, Gongyin Ye

**Affiliations:** 1 State Key Laboratory of Rice Biology, Ministry of Agriculture Key Laboratory of Molecular Biology of Crop Pathogens and Insects, Institute of Insect Sciences, Zhejiang University, Hangzhou, China; 2 USDA/Agricultural Research Service, Biological Control of Insects Research Laboratory, Columbia MO, United States of America; Institut Sophia Agrobiotech, FRANCE

## Abstract

Host plants, pathogens and their herbivore vectors systems have complex relationships via direct and indirect interactions. Although there are substantial gaps in understanding these systems, the dynamics of the relationships may influence the processes of virus transmission and plant disease epidemics. Rice dwarf virus (RDV) is mainly vectored by green rice leafhoppers (GRLHs), *Nephotettix cincticeps* (Uhler) (Hemiptera: Cicadellidae) in a persistently circulative manner. In this study, host plant selection preferences of non-viruliferous and viruliferous (carrying RDV) GRLHs between RDV-free and RDV-infected plants were tested. Non-viruliferous GRLHs preferred RDV-infected rice plants over RDV-free rice plants, and viruliferous GRLHs preferred RDV-free rice plants over RDV-infected rice plants. In odor selection preference bioassay using a four-field olfactometer, non-viruliferous GRLHs preferred odors of RDV-infected rice plants over healthy rice and viruliferous GRLHs preferred odors of RDV-free rice plants over RDV-infected ones. In 6 h plant penetration behavior bioassay using electrical penetration graphs, non-viruliferous GRLHs spent shorter time in non-penetration and much longer time in xylem feeding on RDV-infected, compared to RDV-free rice plants. Viruliferous GRLHs exhibited more salivation and stylet movement on RDV-free rice plants than on RDV-infected rice plants. We infer from these findings that RDV influences these vector behaviors by altering host plant physiology to promote viral transmission.

## Introduction

Rice dwarf virus (RDV), the pathogen causing rice dwarf disease, belongs to Reoviridae, genus *Phytoreovirus* [1]. The RDV genome is composed of 12-segment double-stranded RNAs, packaged within an icosahedral double-shelled particle [2]. It is mainly transmitted by green rice leafhopper (GRLH), *Nephotettix cincticeps* (Uhler) (Hemiptera: Cicadellidae), in a persistently circulative manner and can be transmitted transovarially to their offspring [3, 4]. GRLH acquire the RDV virus particles after feeding on infected plants, for a few minutes to several days. GRLHs are primarily xylem feeders, where they likely acquire most viruses. They can feed on several plant tissues and may acquire viruses from phloem as well. RDVs initially infect the filter chamber epithelium, where progeny virions are assembled. They spread through the digestive system and the hemolymph and finally infect the salivary glands. RDVs infect rice plants while their vector insects ingest plant sap [5, 6].

Host plants, pathogens and their herbivore vectors systems have complex relationships. Plant viruses can influence their vectors, either directly (by infecting vectors) or indirectly (by infecting host plants) [7, 8]. The idea that some plant viruses influence the orientation or settling behavior of their vector insects is very well documented. Wang et al. [9] showed that non-viruliferous white-backed planthoppers *Sogatella furcifera* (Hováth) were more attracted to southern rice black-streaked dwarf virus (SRBSDV)-infected plants than to non-infected plants and, conversely, viruliferous vectors preferred non-infected plants. Several virus-vector-plant systems, such as the barley yellow dwarf virus (BYDV)—*Rhopalosiphum padi* L.—wheat system [10], the potato leaf roll virus (PLRV)—*Myzus persicae—*potato system [11–13] and the tomato spotted wilt virus (TSWV)—*Frankliniella occidentalis* (Pergande)—pepper pathosystems similarly operate [14]. Other vector-virus-host plant systems operate differently. For example, the cucumber mosaic virus (CMV) decreases the quality of its host plant and its aphid vectors (*Myzus persicae* and *Aphis gossypii*) leave infected plants at higher rates, which inhibits prolonged feeding. These aphid behaviors may enhance virus transmission [15, 16]. These publications prompted our question of whether GRLHs have marked preferences for plants that are either infected or not infected with viruses.

Plant viruses may influence vector penetration and feeding behavior, particularly for piercing-sucking insects. Green bugs, *Schizaphis graminum* (Rondani), begin phloem ingestion sooner and invest more time into feeding on BYDV-infected, compared to non-infected oats. They also interrupted feeding bouts less frequently [17]. TSWV-infected western flower thrips, *F*. *occidentalis* (Pergande), made almost 3 times more non-ingestion probes and they salivated during these probes, both prerequisites for virus inoculation, than TSWV-free controls [18]. Tomato Yellow Leaf Curl Virus (TYLCV)-infected whiteflies *Bemisia tabaci* fed more often and had longer salivation phases compared to non-infected whiteflies [19]. Non-infected whiteflies probed faster and had more frequent feeding bouts on TYLCV-infected tomato plants [20]. SRBSDV-infected white-backed planthoppers, *S*. *furcifera* (Horváth), salivated more on non-infected hosts compared to infected hosts and virus-free insects fed more on SRBSDV-infected hosts [21]. We infer that virus infection influences vector penetration and feeding behavior, which may positively influence virus transmission efficiency. The general idea is encapsulated in the “Vector Manipulation Hypothesis” (VMH) [10], although it may not stand up to critical inquiry due to issues such as viral transmission barriers, insect immunity and virus-induced reductions in vector fitness [4]. Because the VMH is not yet settled, more research is necessary to generate broadly applicable principles of virus-insect vector-host plant pathosystems. In this study, we hypothesized that RDV infection influences the orientation and feeding behaviors of its GRLH vector. Here, we report on the outcomes of experiments designed to test our hypothesis.

## Materials and methods

### Leafhopper rearing

The non-viruliferous GRLH population was first collected from experimental rice fields, Zhejiang University, Hangzhou, China, in 2015. The field was founded in 2010 and RDV-infection has not been reported for this site. Stock cultures were maintained on TN1 (Taichung Native 1) seedlings for 3–4 generations within 80-mesh cages (50 cm^3^) in the climate chamber under our standard conditions, 27 ± 1ºC, 75 ± 5% relative humidity, a 14L:10D photoperiod and light intensity of 3, 500–4, 000 lux [22].

To obtain viruliferous GRLHs, non-viruliferous nymphs were confined with RDV-infected TN1 rice seedlings for 2 d (acquisition access period), and then transferred individually to one RDV-free TN1 rice seedling cultured in Kimura solution B [23] in a plastic tube (D = 2.5 cm, H = 25 cm), which was given a reference number. Two days later (inoculation period), each rice seedling was separately transplanted in the glasshouse with its reference number and was replaced with new rice seedlings age 10 ± 2 d for the same leafhopper [22]. Ten days later (virus latent period), the GRLHs were individually collected according to their reference number from plants with characteristic RDV symptoms [24]. GRLHs were separately reared on RDV-infected TN1 plants in a glass tube in a separate climate chamber set under our standard conditions. After emergence, one female and one male were mated in an 80-mesh cage (50 cm^3^) with RDV-infected TN1 plants for oviposition. RDV infection of females was verified by RT-PCR as described below, and F_1_ progeny of viruliferous females were reared together as the viruliferous colony for the experiments.

Numbers of biologically independent replicates are presented in the contexts of each of the three experimental sub-sections. The Statistical Analyses sub-section describes all statistical tests.

### Rice plants

RDV-free and RDV-infected rice seedlings (cv. Xiushui11) were used in our experiments. To obtain RDV-free or RDV-infected rice seedlings, the 10 d old seedlings were individually confined with non-viruliferous or viruliferous fourth-instar GRLH nymph for 2 days and then transplanted into a greenhouse. Thirty days later, RDV-free plants were selected for the experiment. RDV-infected plants were judged by characteristic RDV symptoms [24] and verified by RT-PCR as described below. All plants were cultured in Kimura solution B [23].

### RDV-infection status

The total RNA of vector insects or rice was extracted using TRIzol reagent (Invitrogen, Carlsbad, CA, USA), following the manufacturer’s instructions. The RNA was reverse transcribed into cDNA with the TransScript One-Step gDNA Removal and cDNA Synthesis SuperMix Kit (TransGen Biotech, Beijing) and amplified using primers designed on the RDV S8 fragment [25], forward primer, 5’-CAAAGATCTCCACCTGCCACTATG-3’ (1–24); reverse primer, 5’-GCGCTCGAGATTCAGGACCG-3’ (1378–1397). The amplification program was 94ºC, 3 min; (94ºC, 30 sec; 55ºC, 30 sec; 72ºC, 2 min), 35 cycles; 72ºC, 10 min.

### Host plant selection preference of GRLHs

One RDV-free and one RDV-infected rice seedling were planted in symmetrical spacing 10 cm apart in plastic pots (D = 20 cm), then covered with a transparent polyethylene-plastic cylinder (D = 18 cm, H = 50 cm), with two side-windows (D = 5 cm) of nylon mesh mid-cylinder. The cylinder was covered with nylon mesh at the top for ventilation. Newly emerged non-viruliferous females (*n* = 15; within 24 h after emergence) were fasted for 2 h and transferred into each cage. At 2, 4, 8, 24, 48, 72 and 96 h post-inoculation (PI), the numbers of insects on RDV-free or RDV-infected rice plants were recorded. Ten biologically independent replicates were carried out under our standard conditions in a climate chamber.

To measure the host plant selection preference of viruliferous GRLHs, the experiment was conducted as just described using viruliferous insects.

### Plant odor selection preference of GRLHs

Using a four-field olfactometer (Camsonar SIM-4, Camsonar Group Limited, UK), we recorded the orientation behavioral responses of individual insects to different odors following Faucher et al [26]. The device consisted of a four-pointed star-shaped arena, 1 cm high and 30 cm diagonally. A little piece of sponge prevented GRLHs from entering the nozzle of the four arms. Air was pumped into the arena via the four arms and withdrawn from the central hole in the bottom. Room air was filtered over a glass tube filled with activated carbon, flow-controlled through the rotameter (500 ml min^–1^), then connected to a glass tube (D = 10 cm, H = 55 cm) containing the test materials. An LED lamp (BaDu Lighting, Zhongshan, China, 220V, 50–60 Hz) covered by a piece of frosted glass was installed under the olfactometer such that the arena was illuminated with uniform light to exclude orientation to visual cues.

We set up all the odor materials (healthy rice plants, RDV-infected rice plants, or air) and activated the olfactometer for 10 min before the tests to ensure the arms were filled with odors. A single female, within 24 h after emergence, was fasted for 8 h before the test. Each insect was introduced into the central hole via a small glass tube and each insect was tested only once. We used a digital HD video camera (HDR-PJ610, SONY, Japan) to recorded walking activity and location of each insect for 1 h. The times each insect invested in each field was analyzed using the image processing software (Camsonar Images MP ver1.0, Camsonar Group Limited, UK). GRLHs that did not enter the arena within 10 min were discarded. Ethanol and distilled water were used to wash all equipment thoroughly after 5 runs to avoid accumulation of insect-derived chemicals. Tests were performed in a climate-controlled room at 27 ± 2ºC, RH 70 ± 5% and light intensity of 3, 500–4, 000 lux between 8 a.m. to 8 p.m. At least 15 valid repetitions were obtained for each treatment.

### Treatment groups and electrical penetration graph (EPG) recordings

A Giga-8 direct current EPG (Wageningen University, Wageningen, Netherlands) technique was used to study GRLHs plant penetration behavior [27–29]. Rice seedlings were cultured to 40 d old in Kimura solution B, then transplanted into plastic pots filled with turf soil (D = 5 cm, H = 7 cm) one day in advance for use in this bioassay.

Fourth-instar nymphs (within 24 h after molting) were fasted for 8 h (supplied with water on cotton) before the bioassay. Fourth-instars, whose sex cannot be reliably determined, were selected because the recording set-up interfered with feeding of newly emerged females. Each insect was transferred to a 1.5 ml centrifuge tube and chilled on ice for 1 min to facilitate the attachment of a thin gold wire to the insect. We used a water-soluble silver glue to connect the dorsal thorax of the chilled insect to one end of a gold wire (D = 20 μm, H = 3–4 cm). The glue was dried for about 2 min to ensure a tight junction with the insect. The wired GRLH was connected to the EPG probe via a copper nail and the probe was connected to the amplifier. The test insect was placed on rice leaf surface, noting this attachment did not hamper the insect feeding. A copper wire (D = 2 mm, H = 10 cm) was inserted into the pot soil vertically which was connected to another amplifier as the plant electrode. The electrical EPG signals were digitized with a converter (DI710-UL, Dataq, Akron, USA), and the data acquired and stored with PROBE 3.4 software (Wageningen University, Wageningen, Netherlands) [21]. The substrate voltage was adjusted so that the EPG signals fit into the +5 V to −5 V window provided by the PROBE software [30]. Each insect was continuously recorded for 6 h.

There were two treatments, one with non-viruliferous GRLHs feeding on RDV-free and on RDV-infected plants. The other treatment used viruliferous insects with similar recording of feeding. Each insect and plant was used once. The EPG recordings were conducted in a quiet room at 27 ± 2ºC, RH 70 ± 5% and light intensity of 3, 500–4, 000 lux between 8 a.m. to 8 p.m. We recorded at least 13 biologically independent replicates.

### EPG waveforms and parameters

The EPG waveforms were classified based on Youn [31] and He et al [32]: NP for non-penetration, Nc1 for penetration initiation, Nc2 for salivation and stylet movement, Nc4 for ingestion from phloem bundle tissues, Nc5 for ingestion from xylem bundle tissues.

The period from stylet insertion to stylet withdrawal was defined as a complete probe [21]. During the 6 h experimental period, waveforms were divided into four categories for data analysis: one, durations of each waveform (the time an insect spent in each waveform); two, duration of first NP waveform (the time before stylet inserted to plant vascular tissue); three, probing duration (the sum durations of all the waveforms made by an insect, except NP waveform); and four, probing duration per probe (total probing duration of an insect divided by the number of probes) [21].

### Statistical analysis

Host plant selection preference data were analyzed by the Chi-square test, with null hypothesis of no preference between the treatments (GRLHs that died or were not settled on any plant surface were not included in the statistical analysis) [12]. In odor selection preference assay, to test whether the GRLHs allocated equal time to each field, a nonparametric test was used for dependent data (Friedman-ANOVA, *P* < 0.05) and Wilcoxon-Wilcox as a *post-hoc* test was used to determine whether the time is equally distributed over each field [26]. Tukey’s ANOVA was used for the analysis of non-viruliferous GRLHs and viruliferous GRLHs feeding behavior on RDV-free and RDV-infected rice plants [33]. All statistical analyses were done using the SPSS software (version 16.0) [34] at *P <* 0.05.

## Results

### Host plant selection preference of GRLHs

**Fig 1:**
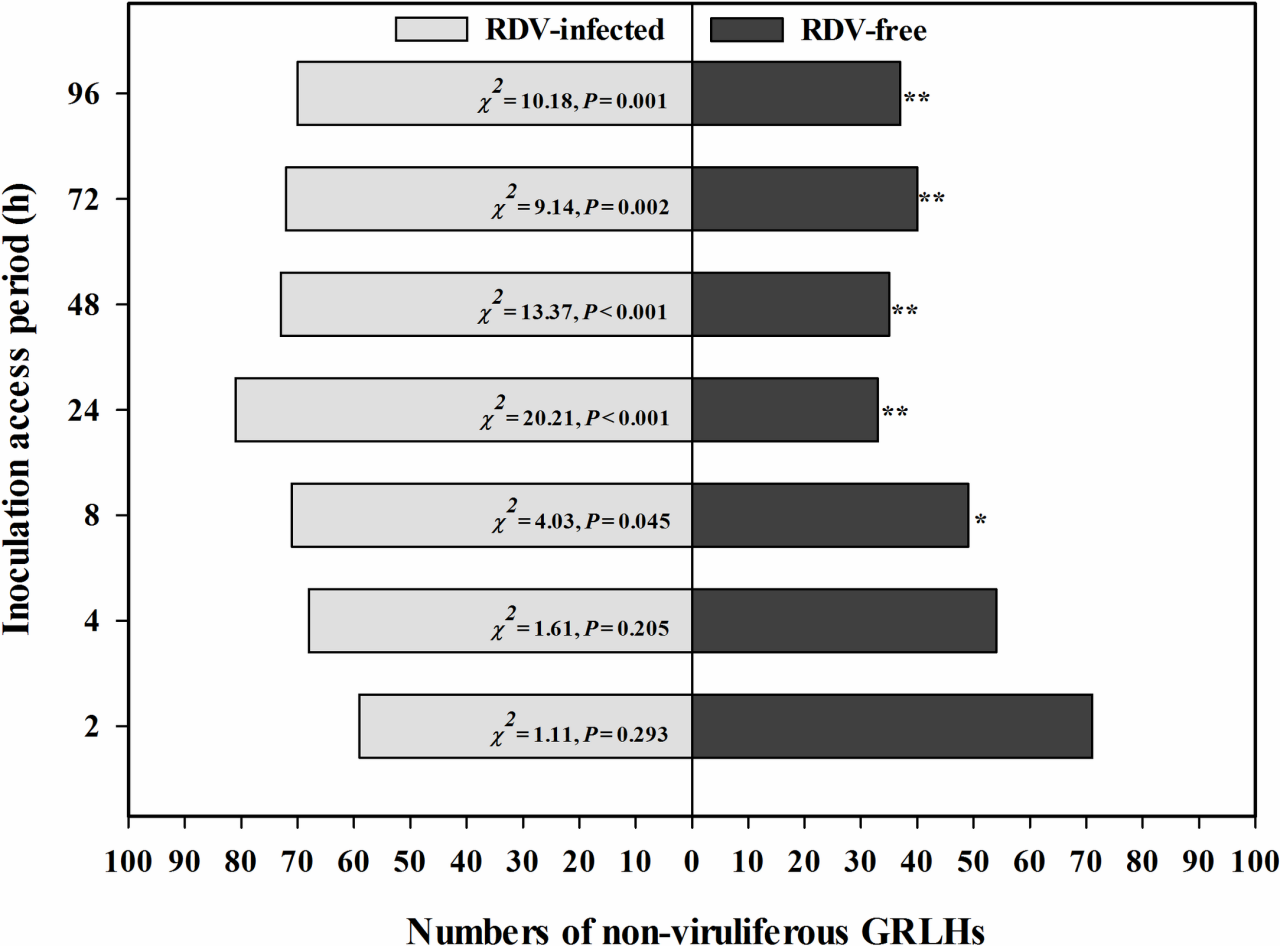
Non-viruliferous GRLHs preferred RDV-infected rice plants over RDV-free rice plants. The histogram bars show numbers of insects settled on RDV-free and RDV-infected rice plants. The asterisks indicate statistical significance, determined by Chi-square test, with null hypothesis of no preference between the treatments (GRLHs not settled on any plant surface or died were not induced in the statistical analysis) [12] (* *P* < 0.05, * * *P* < 0.01).

Host plant selection preferences of non-viruliferous GRLHs between RDV-free and RDV-infected plants were recorded for 4 days after insect release (Fig 1). There were no differences in attraction between the two plant treatments during the first 4 h PI. At 8 h PI, non-viruliferous GRLHs largely preferred to settle on RDV-infected rice plants over RDV-free rice plants (*χ*^*2*^ = 4.03, *P* = 0.045), with about 66% of the insects located on the RDV-infected plants. The non-viruliferous GRLHs had a marked preference for the RDV-infected rice throughout the rest of the re-maining 88 h PI.

**Fig 2:**
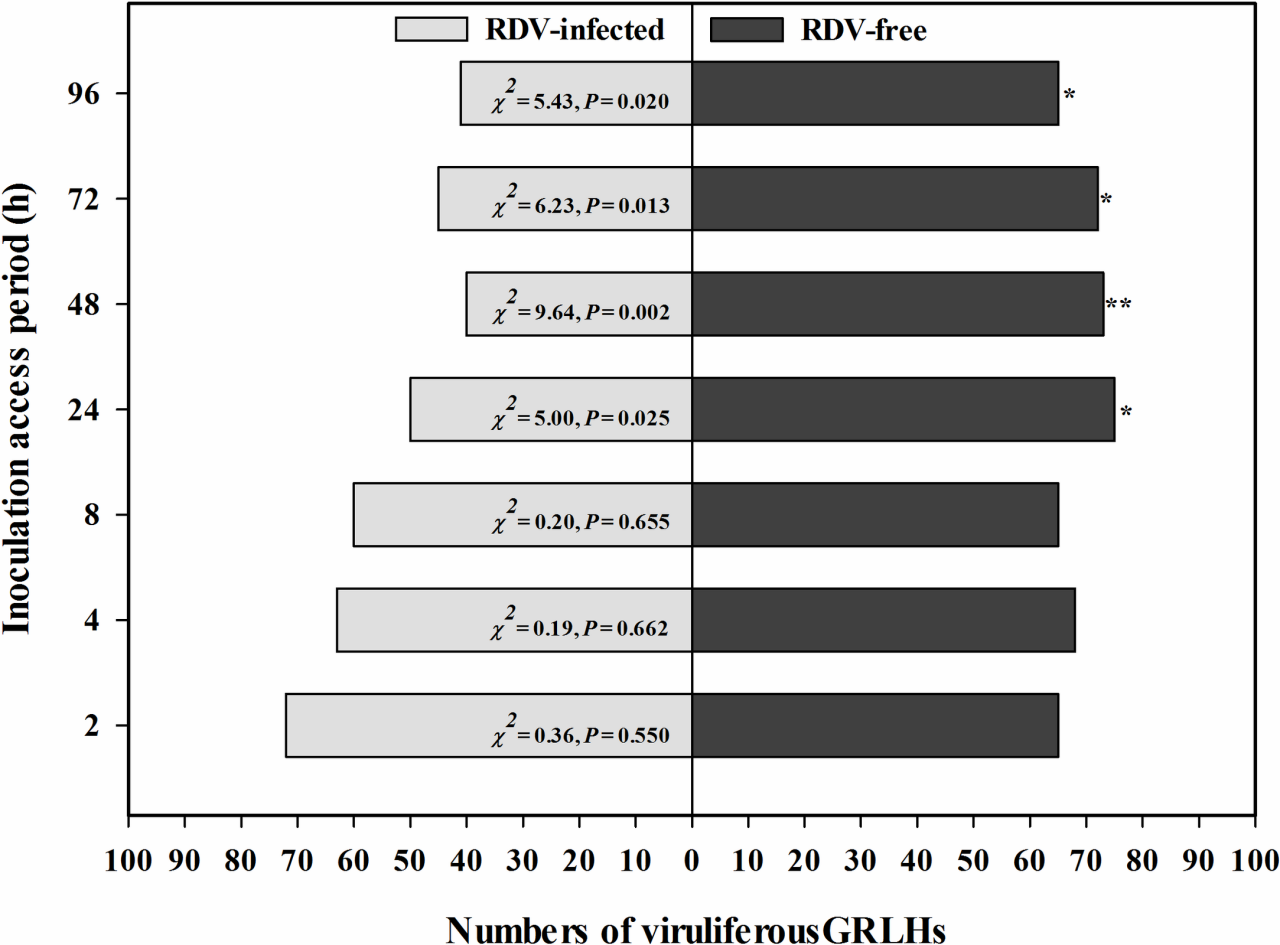
Viruliferous GRLHs preferred RDV-free rice plants over RDV-infected rice plants. The histogram bars show numbers of insects settled on RDV-free and RDV-infected rice plants. The asterisks indicate statistical significance, as determined by the Chi-square test, with null hypothesis of no preference between the treatments (GRLHs not settled on any plant surface or died were not induced in the statistical analysis) [12] (* *P* < 0.05, * * *P* < 0.01).

Viruliferous GRLHs strongly preferred RDV-free plants, compared with the RDV-infected plants (Fig 2). There was no clear selection preference in the initial 24 h PI. The viruliferous GRLHs significantly preferred the RDV-infected plants at 24 h PI (*χ*^*2*^ = 5.00, *P* = 0.025) throughout the following 72 h.

### Plant odor selection preference of GRLHs

**Fig 3:**
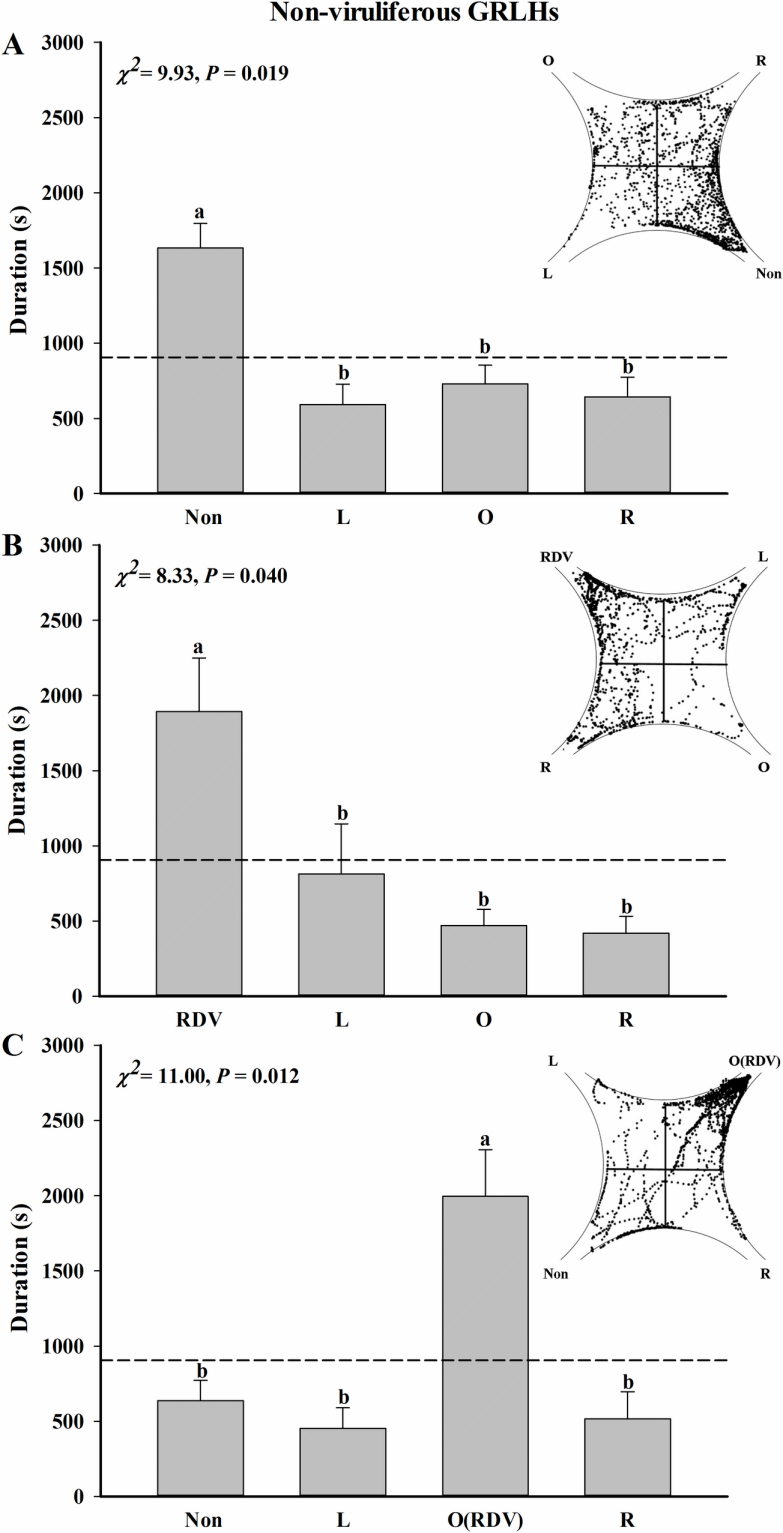
Odor selection preferences of non-viruliferous GRLHs exposed to rice plant volatiles. Preferences were determined as times individual insects invested in each field of a four-field olfactometer during 1 h experiments. (A) RDV-free rice plants were placed in one odor source, with the other three fields charged with filtered clean air (*n* = 18). (B) RDV-infected rice plants were placed in one odor source, with the other three fields charged with filtered clean air (*n* = 15). (C) RDV-free and RDV-infected rice plants were placed in opposite fields (*n* = 20). The orientation of the fields is indicated relative to the field laced with the test odor: Non, RDV-free rice plants; RDV, RDV-infected rice plants; L, left; O, opposite; R, right. Scatter diagram show examples of location of single tracer GRLH per second during a 1 h experiment. The broken line at 900 s indicates an equal amount of time in all fields. Deviations from equal distribution were tested with a Friedman-ANOVA (*P* < 0.05). Bars annotated with different letters were significantly different from each other (Wilcoxon-Wilcox test).

When the odor of non-infected rice plants was added to one field, the non-viruliferous GRLHs walked almost 45% time in this field, significantly more than the other three filtered air control fields (Fig 3A, *n* = 18, *χ*^*2*^ = 9.93, *P* = 0.019). The GRLHs were attracted to the test field (RDV-infected plant volatiles), where they invested more time compared to the three filtered air control fields (Fig 3B, *n* = 15, *χ*^*2*^ = 8.33, *P* = 0.040). After they were exposed to two odors simultaneously in the opposite fields, the non-viruliferous GRLHs invested 1994.9 sec in the field charged with RDV-infected rice plants, almost 3.1-fold more than the duration of invested in the field charged with non-infected rice plants (Fig 3C, *n* = 20, *χ*^*2*^ = 11.00, *P* = 0.012).

**Fig 4:**
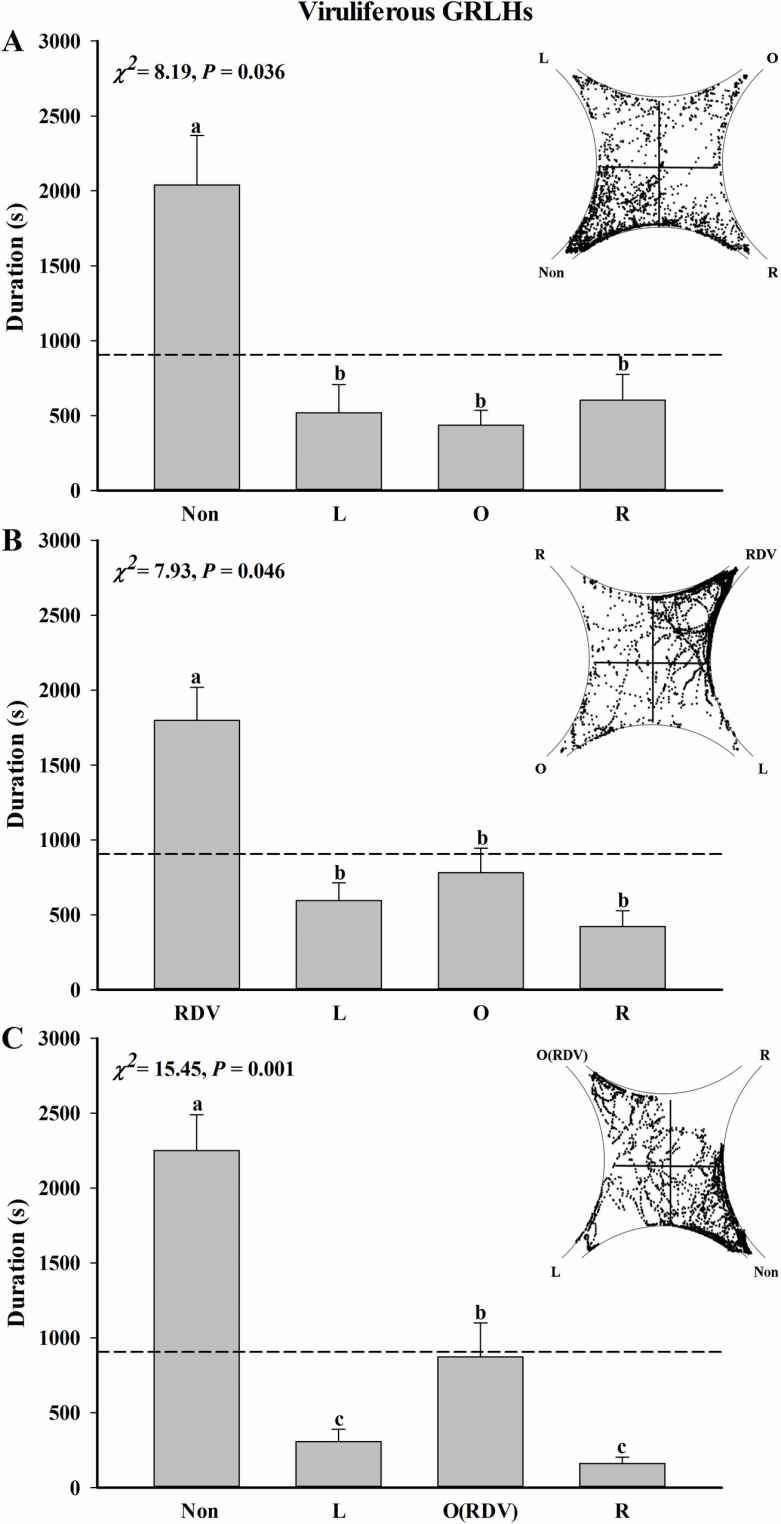
Odor selection preferences of viruliferous GRLHs exposed to rice plant volatiles. Preferences were determined as times individual insects invested in each field of a four-field olfactometer during 1 h experiments. (A) Same as Fig 3 panel A (*n* = 17). (B) Same as Fig 3 panel B (*n* = 18). (C) Same as Fig 3 panel C (*n* = 20). Scatter diagram show examples of location of single tracer GRLH per second during a 1 h experiment. The broken line at 900 s indicates an equal amount of time in all fields. Deviations from equal distribution were tested with a Friedman-ANOVA (*P* < 0.05). Bars annotated with different letters are significantly different from each other (Wilcoxon-Wilcox test).

The odor selection preferences of viruliferous GRLHs were also determined. Compared with control fields charged with filtered room air, the viruliferous GRLHs were attracted to the odor of non-infected rice plants (Fig 4A, *n* = 17, *χ*^*2*^ = 8.19, *P* = 0.036), and, separately, RDV-infected rice plants (Fig 4B, *n* = 18, *χ*^*2*^ = 7.93, *P* = 0.046). Behavioral response of the viruliferous GRLHs to non-infected rice plants and RDV-infected rice plants indicated that odor of non-infected rice plants is more attractive to the test insects than RDV-infected rice plants (Fig 4C, *n* = 20, *χ*^*2*^ = 15.45, *P* = 0.001).

### Plant penetration behavior of GRLHs by EPG

Five distinctive waveforms were recorded during probing by the GRLH as seen in other reports [30], and no new waveforms were recorded (S1 Fig).

**Fig 5:**
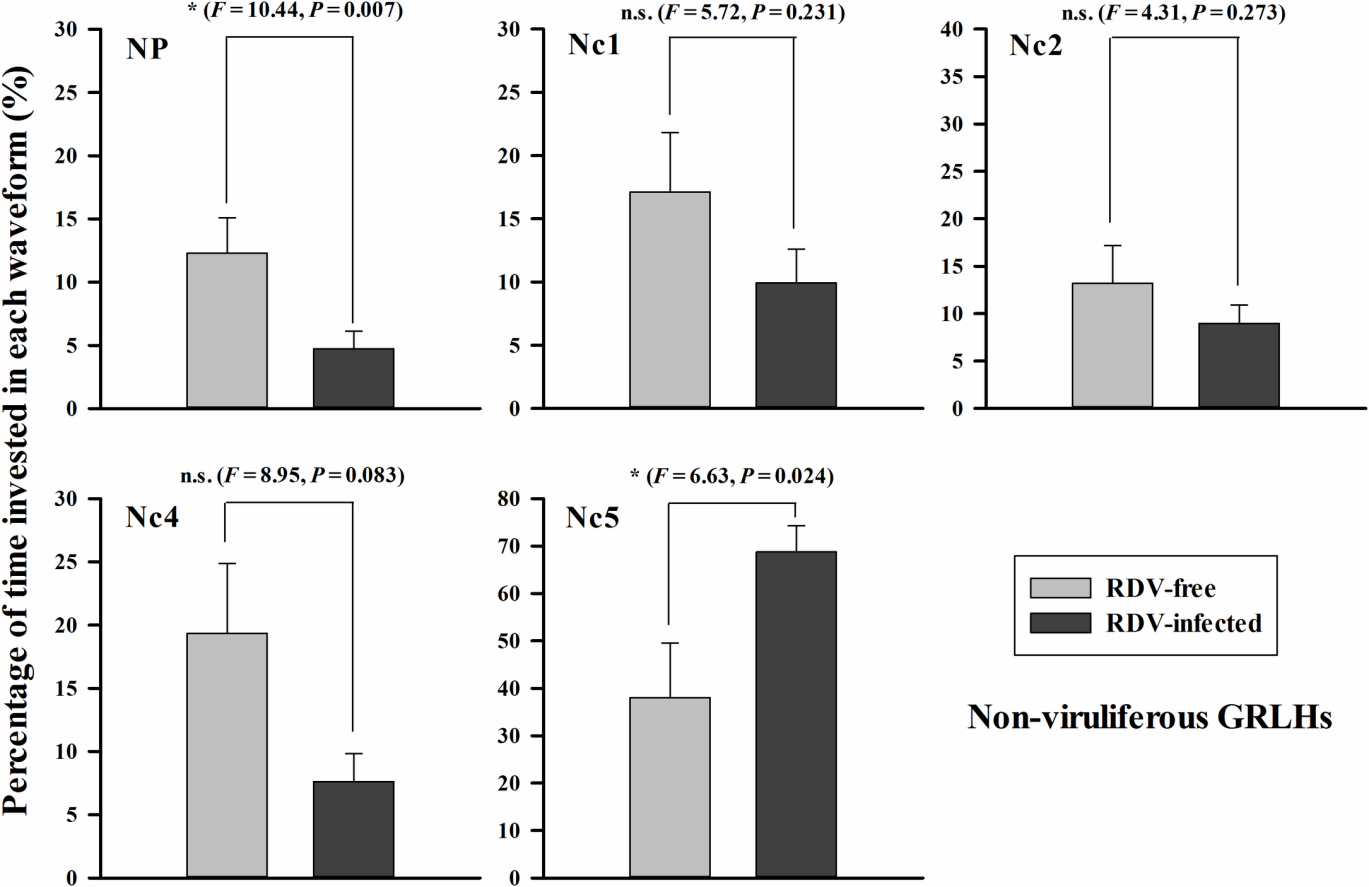
Non-viruliferous GRLHs invested time in each waveform during 6 h recording periods. The histogram bars show percentages of time invested in each waveform on RDV-free (*n* = 13) and RDV-infected (*n* = 15) rice plants. NP = non-penetration, Nc1 = penetration initiation, Nc2 = salivation and stylet movement, Nc4 = ingestion from phloem bundle tissues, Nc5 = ingestion from xylem bundle tissues. The data are means ± s. e. m. Histogram bars annotated with * indicate significant difference (*P* < 0.05), ns indicates no significant difference (*P* > 0.05).

With non-viruliferous GRLH, we obtained 13 biologically independent replicates on RDV-free plants and 15 on RDV-infected plants (Fig 5). During the 6 h recording periods, non-viruliferous GRLH invested varying proportions of their time in all waveforms on RDV-free plants, NP (12.3 ± 2.8%), Nc1 (17.1 ± 4.7%), Nc2 (13.2 ± 4.0%), Nc4 (19.4 ± 5.5%) and Nc5 (38.0 ± 11.5%). The proportions were otherwise on RDV-infected plants, NP (4.7 ± 1.4%), Nc1 (9.9 ± 2.7%), Nc2 (9.0 ± 1.9%), Nc4 (7.6 ± 2.2%) and Nc5 (68.7 ± 5.6%). Overall, non-viruliferous insects spent shorter time in non-penetration (NP) (*F* = 10.44, *P* = 0.007), almost 2-fold longer time in xylem (Nc5) (*F* = 6.63, *P* = 0.024) on RDV-infected compared with RDV-free rice plants. There were no significant differences among the other three waveforms.

**Fig 6:**
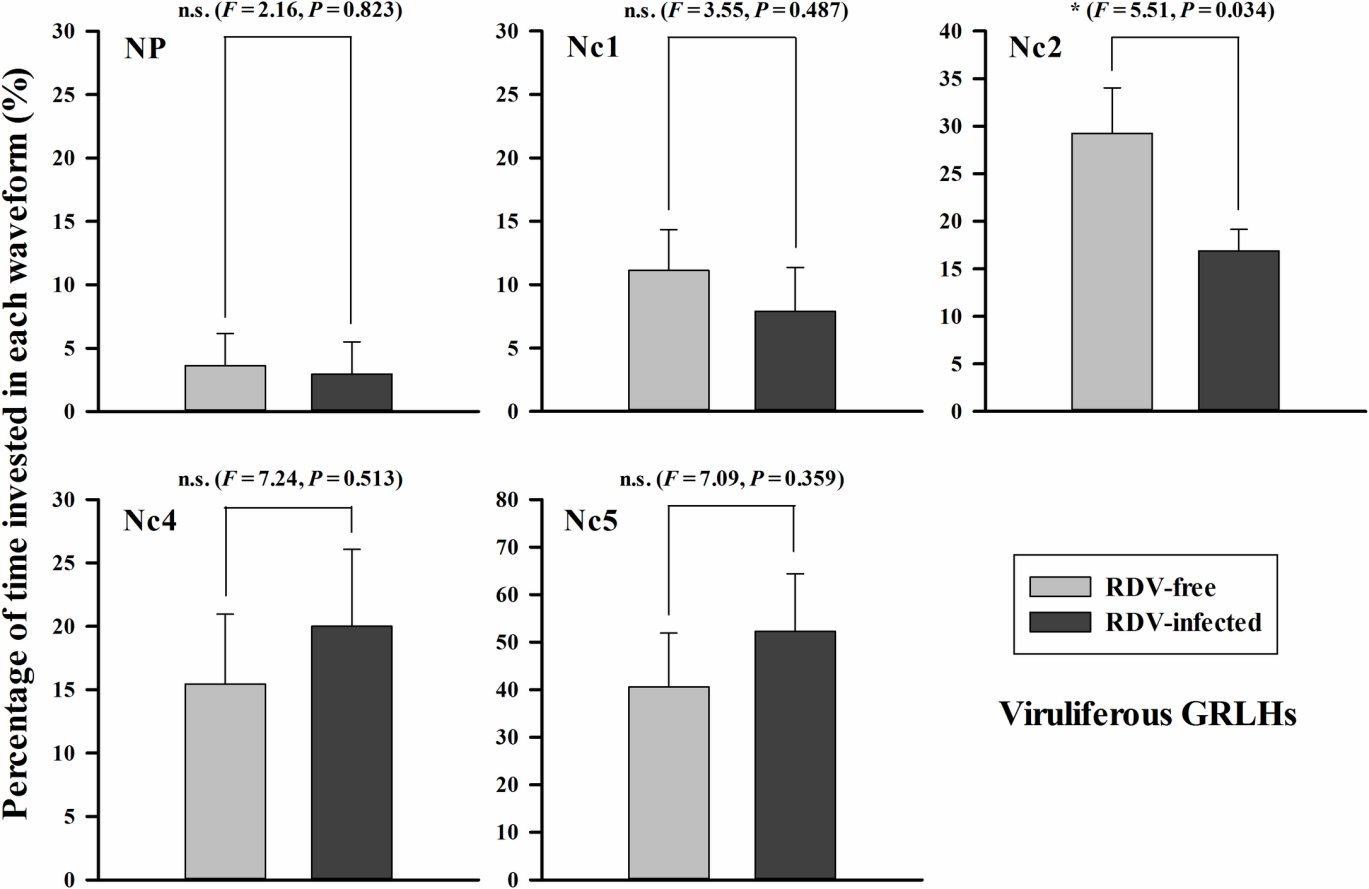
Viruliferous GRLHs invested time in each waveform during 6 h recording periods. The histogram bars show percentage of time invested in each waveform on RDV-free (*n* = 14) and RDV-infected (*n* = 17) rice plants. The data are means ± s. e. m. Histogram bars annotated with * indicate significant differences (*P* < 0.05), ns indicates no significant difference (*P* > 0.05).

With viruliferous GRLH, we obtained 14 biologically independent replicates on RDV-free plants and 17 on RDV-infected plants (Fig 6). Again, the insects invested different time periods in each of the waveforms. For RDV-free plants, the proportions of time in each waveform were NP (3.6 ± 2.5%), Nc1 (11.1 ± 3.2%), Nc2 (29.3 ± 4.8%), Nc4 (15.4 ± 5.5%) and Nc5 (40.6 ± 11.3%). On RDV-infected plants, corresponding parameters were NP (3.0 ± 2.5%), Nc1 (7.9 ± 3.5%), Nc2 (16.9 ± 2.3%), Nc4 (20.0 ± 6.1%) and Nc5 (52.3 ± 12.1%). Only the percentage of salivation and stylet movement (Nc2) were significantly different between non-infected plants and RDV-infected plants (*F* = 5.51, *P* = 0.034). Viruliferous GRLHs spent more than 1.7-fold time on salivation and stylet movement (Nc2) on non-infected rice plants.

**Fig 7:**
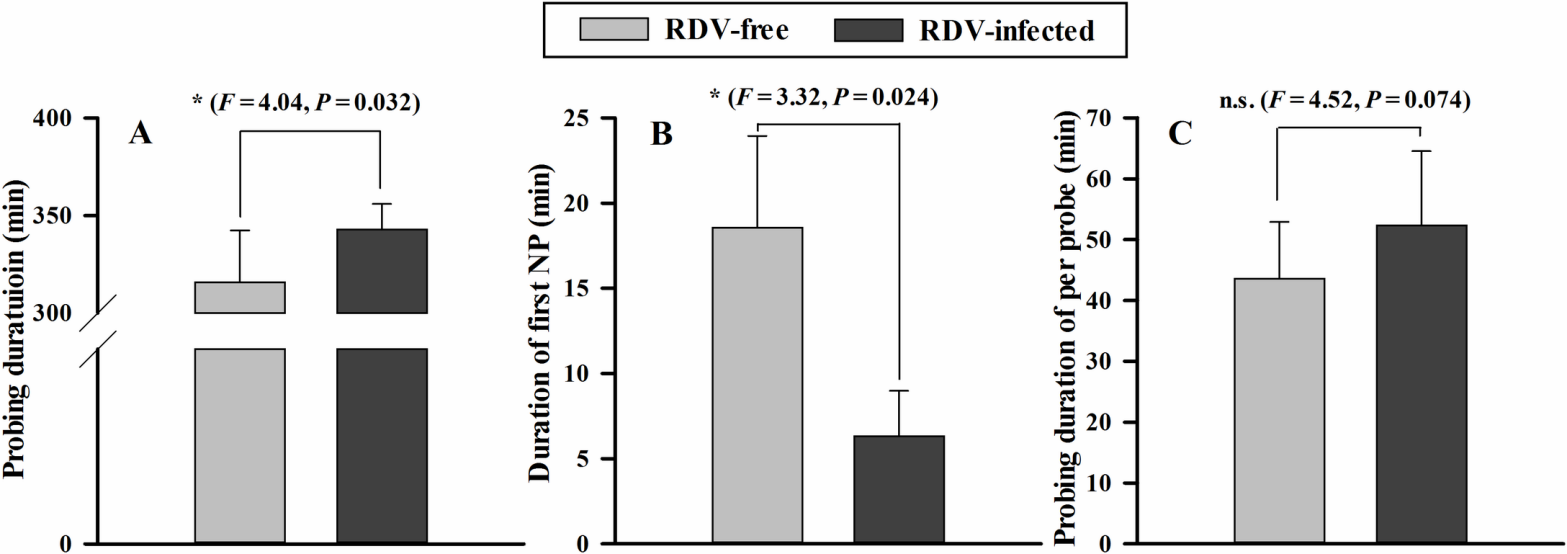
Three EPG parameters of non-viruliferous GRLHs feeding on RDV- free (*n* = 13) and RDV-infected (*n* = 15) rice plants. The data were electrically recorded during 6 h feeding periods on rice plants. The histogram bars show time (min) spent on each of the rice treatments. The data are means ± s. e. m. Histogram bars annotated with * were significantly different (*P* < 0.05).

**Fig 8:**
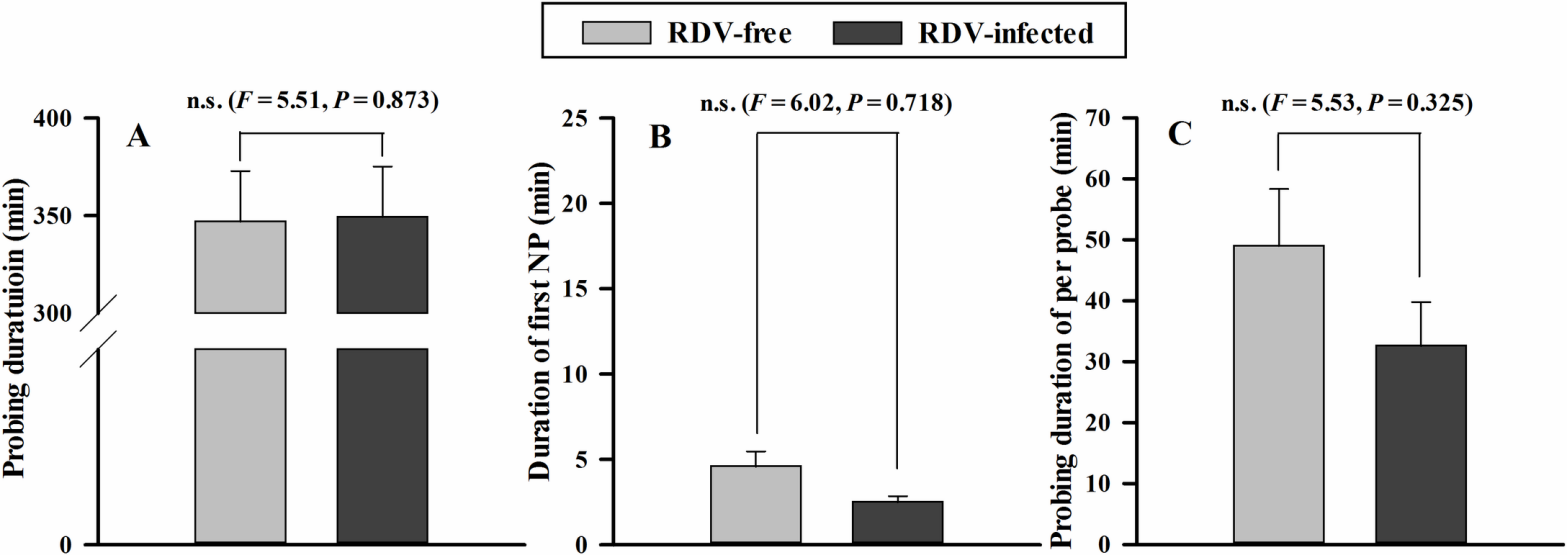
Three EPG parameters of viruliferous GRLHs feeding on RDV- free (*n* = 14) and RDV-infected (*n* = 17) rice plants. The data were electrically recorded during 6 h feeding periods on rice plants. The histogram bars show time (min) spent on each of the rice treatments. The data are means ± s. e. m. Histogram bars annotated with * were significantly different (*P* < 0.05).

The non-viruliferous GRLHs invested more time in probe duration per insect on RDV-infected plants (Fig 7A, *F* = 4.04, *P* = 0.032) and less time in first NP per insect (Fig 7B, *F* = 3.32, *P* = 0.024). On RDV-infected plants, the probe durations per probe were similar on both plant types (Fig 7C, *F* = 4.52, *P* = 0.074). These parameters were similar for viruliferous GRLH, with no significance differences in probe duration per insect (Fig 8A, *F* = 5.51, *P* = 0.873), duration of first NP per insect (Fig 8B, *F* = 6.02, *P* = 0.718), or probe duration per probe (Fig 8C, *F* = 5.53, *P* = 0.325).

## Discussion

Plant viruses have complex relationships with their insect vectors and host plants. In the tritrophic virus-vector-plant relationships, plant viruses can influence their vector’s feeding, development, fitness and reproduction, either directly (by infection of vector) or indirectly (by infection of host plants) [7, 8]. Our data convincingly support our hypothesis that RDV infection influences the behaviors of its GRLH vector. Several points are germane. First, non-viruliferous GRLHs had a marked selection preference for RDV-infected plants over their RDV-free counterparts and, conversely, viruliferous GRLHs preferred RDV-free rice plants over RDV-infected plants. Second, non-viruliferous GRLHs preferred odors of RDV-infected rice plants over RDV-free plants and viruliferous GRLHs preferred odors of RDV-free plants over RDV-infected ones. Third, RDV infection influenced the penetration and feeding behavior of the tested insects. Taken together, these points document the influence of viral infection status of the host plants on the vector insect behavior.

The central point of the VMH is that parasites or their offspring must somehow move from one host to the next. The idea that parasites somehow manipulate their hosts or vectors to enhance the moving process has been in the literature for decades and it covers parasites of vertebrates and a wide range of invertebrates [35]. Plant pathogenic viruses change the physiology of their host plants, altering the volatile chemicals they emit and, hence odors, and also changing their spectral properties, which may change visual cues received by vectors [36]. In their work with aphids and barley yellow dwarf virus, Ingwell [10] show that virus-infected wheat plants are more attractive to non-infected aphids and that non-infected plants are more attractive to infected aphids. Our data similarly show that virus-infected rice plants emit odors that differ from non-infected plants. The core understanding is that parasites are able to alter the phenotypes of their host plants and insect vectors are able to detect the altered phenotypes and make choices that depend on their own infection status. Our working hypothesis is the vector insects are influenced by plant volatile organic compounds, a point now under investigation. We infer that viral infection influences host plant physiology and vector insect behavioral physiology.

Orientation or, settling behavior of vector insects, is influenced by plant viruses. Such behavior may promote pathogen acquisition and transmission, as noted in the Introduction [9–16]. The specific mechanisms of influencing vector behaviors are largely unknown, although viral infection may operate to change host plant physiology in a way that alters the composition of plant fluids and volatile organic compounds [15, 16, 37]. We suspect many viral mechanisms operate to influence their host plants. For a single example, *Nicotiana benthamiana* (a tobacco relative) and the model plant *Arabidopsis thaliana* are host plants for the Turnip Mosaic Virus (TMV). TMV-infected plants promote growth and reproduction of its aphid vector, *Myzus persicae*. Casteel et al. [38] reported that over-expression of a single viral protein, nuclear inclusion a-protease domain (Nla-Pro), is sufficient to increase aphid growth and reproduction. More recently, they reported that the viral Nla-Pro changes its intracellular localization within host plant cells to the vacuole in the presence of aphids, which is required to promote aphid performance [39]. We infer that plant viruses have evolved mechanisms to create fundamental changes in host plant physiology.

RDV infection influenced GRLH penetration and feeding behavior. Non-viruliferous GRLHs spent shorter times in NP and more time in xylem feeding. These behaviors may enhance RDV acquisition. Viruliferous GRLHs spent more time in Nc2 on RDV-free plants, possibly promoting virus transfer to the plants. Reports of similar studies are consistent with our findings [17–21]. Virus inoculation leads, also, to changes in systemic plant physiology, including compositions of amino acids, carbohydrate, and other nutrients of plant fluids. Such changes have been confirmed to influence insect probing and feeding behavior and they may contribute to virus acquisition and transmission [40–43].

Pathogenic organisms have co-evolved with their host plants and vectors, affecting the performance and behavior of vectors in ways that seem to favor their spread and transmission [44–47]. In the present study, host preference bioassay and feeding behavior details indicate that GRLH probing and feeding behavior were manipulated by the virus to favor itself. Generally, our findings support the hypothesis that RDV infection influences the orientation and feeding behaviors of its GRLH vector, impacting transmission and acquisition of RDV. We propose these virus-driven changes in vector insect behavior supports the VMH, as discussed just above [10]. Although the idea has not been directly tested in this work, reports indicate that virus populations and biogeography are largely determined by activity and behavior of vector insects [48, 49]. The biological significance of pathogen-influenced vector behaviors may lead to increased transmission and geographical ranges of pathogens. In our view, in the very extensive plant monocultures associated with modern agro-ecosystems, very small insects, such as planthoppers, aphids and whiteflies, require only small movements to spread pathogens. Here, we emphasize that long-term insect vector monitoring and management are necessary to minimize plant disease epidemics.

## Supporting information

S1 Fig Characteristic electrical penetration graph waveforms recorded from green rice leafhoppers (GRLHs) feeding on rice plants. (A) 100s recording of waveforms NP, Nc1 and Nc2; (B) 60s recording of waveforms Nc4; (C) 10s amplification of Nc4; (D) 60s recording of waveforms Nc5; (E) 10s amplification of Nc5. NP = non-penetration, Nc1 = penetration initiation, Nc2 = salivation and stylet movement, Nc4 = ingestion from phloem bundle tissues, Nc5 = ingestion from xylem bundle tissues.
(TIF)
