# Supplementary figures and images for: Rice dwarf virus infection alters green rice leafhopper host preference and feeding behavior
Source: PLoS One. 2018 Sep 7;13(9):e0203364. doi: 10.1371/journal.pone.0203364 (PMC6128522; doi:10.1371/journal.pone.0203364)

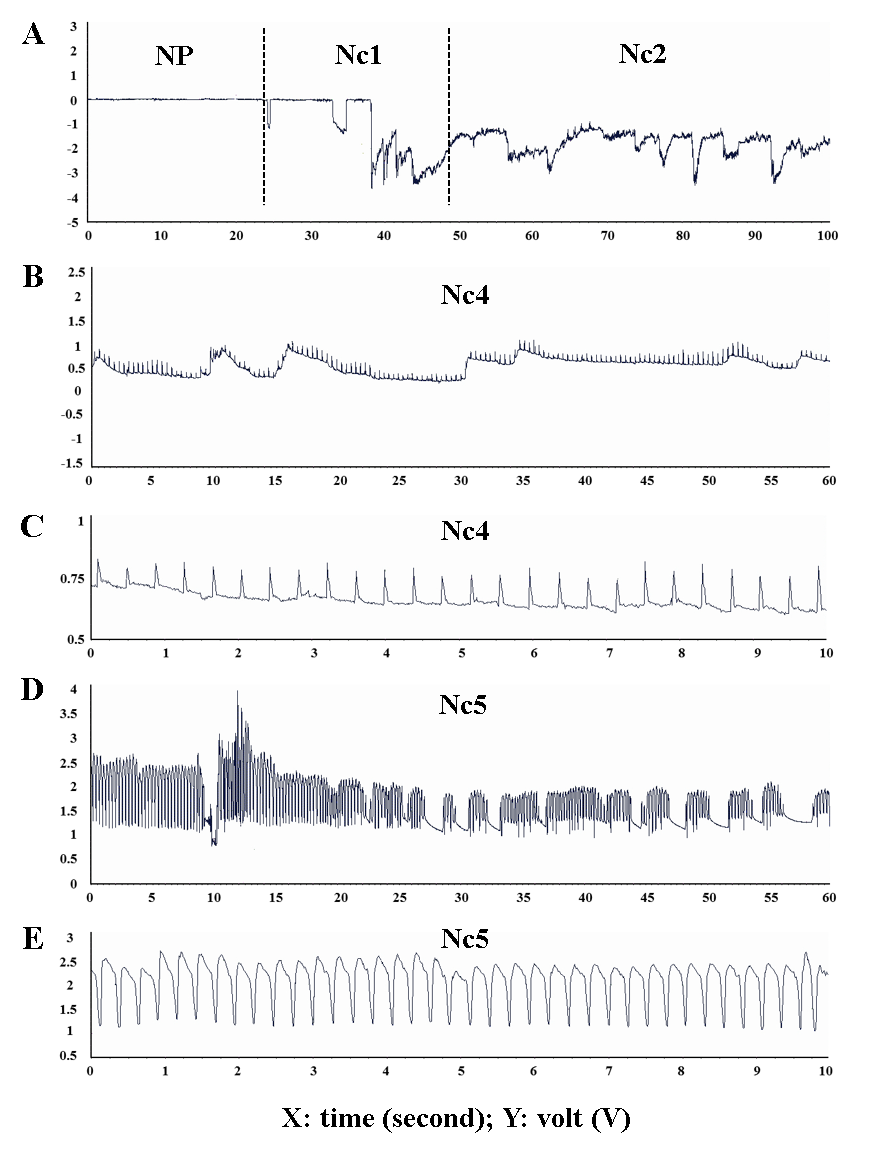

Supplement: S1 Fig — (A) 100s recording of waveforms NP, Nc1 and Nc2; (B) 60s recording of waveforms Nc4; (C) 10s amplification of Nc4; (D) 60s recording of waveforms Nc5; (E) 10s amplification of Nc5. NP = non-penetration, Nc1 = penetration initiation, Nc2 = salivation and stylet movement, Nc4 = ingestion from phloem bundle tissues, Nc5 = ingestion from xylem bundle tissues. (TIF) [file pone.0203364.s001.tif]
